# Fully-automated root image analysis (faRIA)

**DOI:** 10.1038/s41598-021-95480-y

**Published:** 2021-08-06

**Authors:** Narendra Narisetti, Michael Henke, Christiane Seiler, Astrid Junker, Jörn Ostermann, Thomas Altmann, Evgeny Gladilin

**Affiliations:** 1 Leibniz Institute of Plant Genetics and Crop Plant Research, OT Gatersleben, Corrensstr. 3, 06466 Seeland, Germany; 2 Institute for Information Processing (TNT), Leibniz University of Hannover, Appelstr. 9A, 30167 Hannover, Germany; 3 Present Address: Plant Sciences Core Facility, CEITEC-Central European Institute of Technology, Masaryk University, 62500 Brno, Czech Republic

**Keywords:** Image processing, Software, Plant sciences

## Abstract

High-throughput root phenotyping in the soil became an indispensable quantitative tool for the assessment of effects of climatic factors and molecular perturbation on plant root morphology, development and function. To efficiently analyse a large amount of structurally complex soil-root images advanced methods for automated image segmentation are required. Due to often unavoidable overlap between the intensity of fore- and background regions simple thresholding methods are, generally, not suitable for the segmentation of root regions. Higher-level cognitive models such as convolutional neural networks (CNN) provide capabilities for segmenting roots from heterogeneous and noisy background structures, however, they require a representative set of manually segmented (ground truth) images. Here, we present a GUI-based tool for fully automated quantitative analysis of root images using a pre-trained CNN model, which relies on an extension of the U-Net architecture. The developed CNN framework was designed to efficiently segment root structures of different size, shape and optical contrast using low budget hardware systems. The CNN model was trained on a set of 6465 masks derived from 182 manually segmented near-infrared (NIR) maize root images. Our experimental results show that the proposed approach achieves a Dice coefficient of 0.87 and outperforms existing tools (e.g., SegRoot) with Dice coefficient of 0.67 by application not only to NIR but also to other imaging modalities and plant species such as barley and arabidopsis soil-root images from LED-rhizotron and UV imaging systems, respectively. In summary, the developed software framework enables users to efficiently analyse soil-root images in an automated manner (i.e. without manual interaction with data and/or parameter tuning) providing quantitative plant scientists with a powerful analytical tool.

## Introduction

Image based high-throughput phenotyping of roots is one of the emerging disciplines in plant phenomics. It aims to extract the plant morphological and physiological properties in a non-destructive manner to study the plant performance under given conditions^1^. Traditional approaches to root phenotyping have relied on destructive and artificial grown mediums such as liquids or gels^2,3^. However, the root growth is known to be dependent on physical conditions^4^ and such studies have shown a non-typical response of the roots in soil^5,6^.

More recently, non-destructive methods such as X-ray computed tomography^7,8^, nuclear magnetic resonance (NMR) microscopy^9^ and laser scanning^10^ provide unique insights into 3D organization of living root architecture, however, their throughput capabilities are presently rather limited. Moreover, minirhizotrons^11,12^ and rhizotron systems^13,14^ have gained popularity to enable non-invasive imaging of roots in a soil environment. However, the minirhizotrons require a repeated photographing of roots through a transparent surface of below ground observation tubes^15^. In contrast, rhizotron systems contain rectangular glass pots which requires a single photographing of roots^16^. Recently, near-infrared (NIR) imaging of roots growing along transparent pots were presented in our previous works^17,18^. These systems contain special low pass filters to block root exposure to visible light and the images were taken by NIR camera under suitable illumination.

Due to high level of optical soil heterogeneity, soil-root images exhibit a relatively low contrast between back- and foreground structures. Consequently, at the local scale root and soil pixels cannot be distinguished on the basis of their intensity values only. Several root image solutions were suggested in the past, however, most of them were designed for a specific imaging system^19–23^. Examples of general-purpose semi-automated tools include GiA Roots^24^, IJ-Rhizo^25^ as well as our previously published saRIA software^26^. All these tools rely on thresholding and morphological filtering techniques to segment the roots from background. Other root phenotyping solutions like SmartRoot^27,28^ require manual segmentation by placing multiple landmarks along the roots that are subsequently interconnected to the root skeleton. All the above software solutions are time consuming, have limited throughput capabilities, and require expertise in parameter tuning.

To overcome the limitations of existing methods, automated root image segmentation solutions are required for high-throughput root image segmentation and phenotyping. In the last 5 years, deep learning gained high attention especially in computer vision applications, because of the ability to directly extract and train relevant multi-level features from data without prior knowledge and human effort in feature design. Convolutional neural networks (CNNs) are a class of deep learning approaches that have shown to outperform traditional methods in many applications of the computer vision that are associated with higher level cognitive abilities^29^. CNNs have been shown to outperform conventional approaches when applied to traditionally difficult tasks of image analysis including pattern detection and object segmentation in biomedical images^30,31^, traffic scenes^32^ and remote sensing^33^. In recent years, they were also used for high-throughput plant phenotyping such as detection of wheat roots grown in germination paper^34^, segmentation of roots from soil in X-ray tomography^35^ and segmentation of spikes in wheat plants^36^. However, most of these works present exemplary application and/or computational frameworks that can hardly be handled by end-users without advanced programming skills.

The focus of this work is on semantic segmentation of soil-root images by which root pixels are automatically segmented from soil regions. For this kind of approach, CNNs often use encoder–decoder architecture. Till date, several papers have been published on this type of CNN architecture for biomedical^30,31^ and areal applications^32,33^. Moreover, this type of architectures are constantly improving by cascading or fusing the CNNs in biomedical^37,38^ and remote sensing applications^39^.

Application of CNNs to automated image analysis and plant phenotyping became an emerging trend in quantitative plant sciences in the recent years^40^. However, reliable software tools suitable for a particular plant type are rarely available due to the large variability of optical plant appearance, differences between experimental setups^35,40^, and the absence of labelled ground truth data^41,42^. Consequently, only a few software tools for high-throughput plant image analysis and phenotyping are presently known.

Previously published state of the art encoder–decoder CNN solutions for root image segmentation include RootNav 2.0^43^, SegRoot^44^ and RootNet^45^. Among those, RootNav 2.0 and RootNet tools were primarily developed for particular experimental setups such as roots grown on germination paper with high contrast between root and (blue) background pixels, and, thus, cannot be expected to perform accurately by application to other imaging modalities such as noisy soil-root images in this work.

Among the above mentioned tools, SegRoot appears to be the most suitable one for soil-root image segmentation as it is previously shown to be capable of segmenting roots from soil background in minirhizotrons systems. Moreover, the architecture of SegRoot is somewhat similar to U-Net and it transfers the location of feature maps to decoder for image segmentation. However, this approach failed to detect fine, blurry and low contrast roots, which, in turn, compromises the accuracy of resulting phenotypic traits such as estimated root biomass and other geometric features. To overcome these limitations, here, we adopted a U-Net^30^ based encoder–decoder architecture which transfers both location and pixel information of the feature maps to the decoder. Also, it is especially useful when large amount of manually annotated data is challenging, such as often the case in biomedical applications.

The aim of this work is to develop an efficient and handy tool for fully automated root image segmentation and quantification using a pre-trained deep CNN framework which could be used in a straightforward manner even by unskilled users. Although, our approach relies on supervised model training, for the end-users such a model-based image analysis is performed in a fully automated manner (i.e. without interaction with data and/or parameter tuning) in contrast to purely manual or semi-automated image segmentation approaches where such interactions are required. Consequently, we termed this approach fully-automated root image analysis (faRIA). The main contributions of this work include:

- Development of a CNN approach to automated root image segmentation based on the U-Net architecture from^30^,
- Training and application of the CNN model for efficient segmentation of root structures of different size, shape and optical contrast on low budget hardware systems using image masking approach,
- Evaluation and comparison of our CNN model vs. other state-of-the-art tools for root image analysis using the Dice similarity metrics,
- Evaluation of our CNN framework performance on images of different root imaging modalities,
- Development of a GUI based front-end for efficient handling of the algorithmic framework suitable also for IT-unskilled users.

The paper is structured as follows: first, we describe the methodological framework of proposed U-Net based deep learning algorithm and performance matrices for soil-root segmentation. Then, a brief experimental setup consist of data preparation, training and prediction procedure are discussed. Followed by, the results of experimental investigation are presented including a comparison of faRIA performance to other image segmentation tools, performance on resized images and robustness by application to other image modalities and plant species. In discussion, we summarize the results of an evaluation study using faRIA image segmentation and present its GUI implementation for efficient application in high-throughput root phenotyping.

## Methods

### Deep CNN model for root image segmentation

The proposed CNN architecture is derived from the original U-Net^30^ which provides a versatile framework for semantic image segmentation consisting of encoder and corresponding decoder units. Our CNN model has a depth of 3 which is less than original U-Net depth of 4 due to the smaller input image size. Further, in our approach batch normalization^46^ is applied after each convolutional layer in contrast to the original U-Net architecture where it was not the case. The motivation behind the batch normalization is it is known to make model performance more faster and stable^46,47^. Furthermore, the original U-Net^30^ used dropout layer which we avoided because in some cases the combination of batch normalization and dropout layers can cause worse results^48^. Also, kernel size of the convolutional layers was set larger in our approach than in the original U-Net to improve the continuity in segmentation of roots^49^. The details of the convolutional parameters in comparison to the original U-Net are summarized in Table 1.

**Table 1:** Convolutional parameters of the original U-Net and proposed modifications.

Convolutional parameters	Original U-Net	Proposed modifications
Kernel size	$3\times 3$	$7\times 7$
Transposed kernel size	$2\times 2$	$3\times 3$
Stride	$1\times 1$	$2\times 2$
Padding	Unpadded	Padding with zeros
Depth	4	3
Number of filters	(64, 128, 256, 512, 1028)	(16, 32, 64, 128)

**Figure 1:**
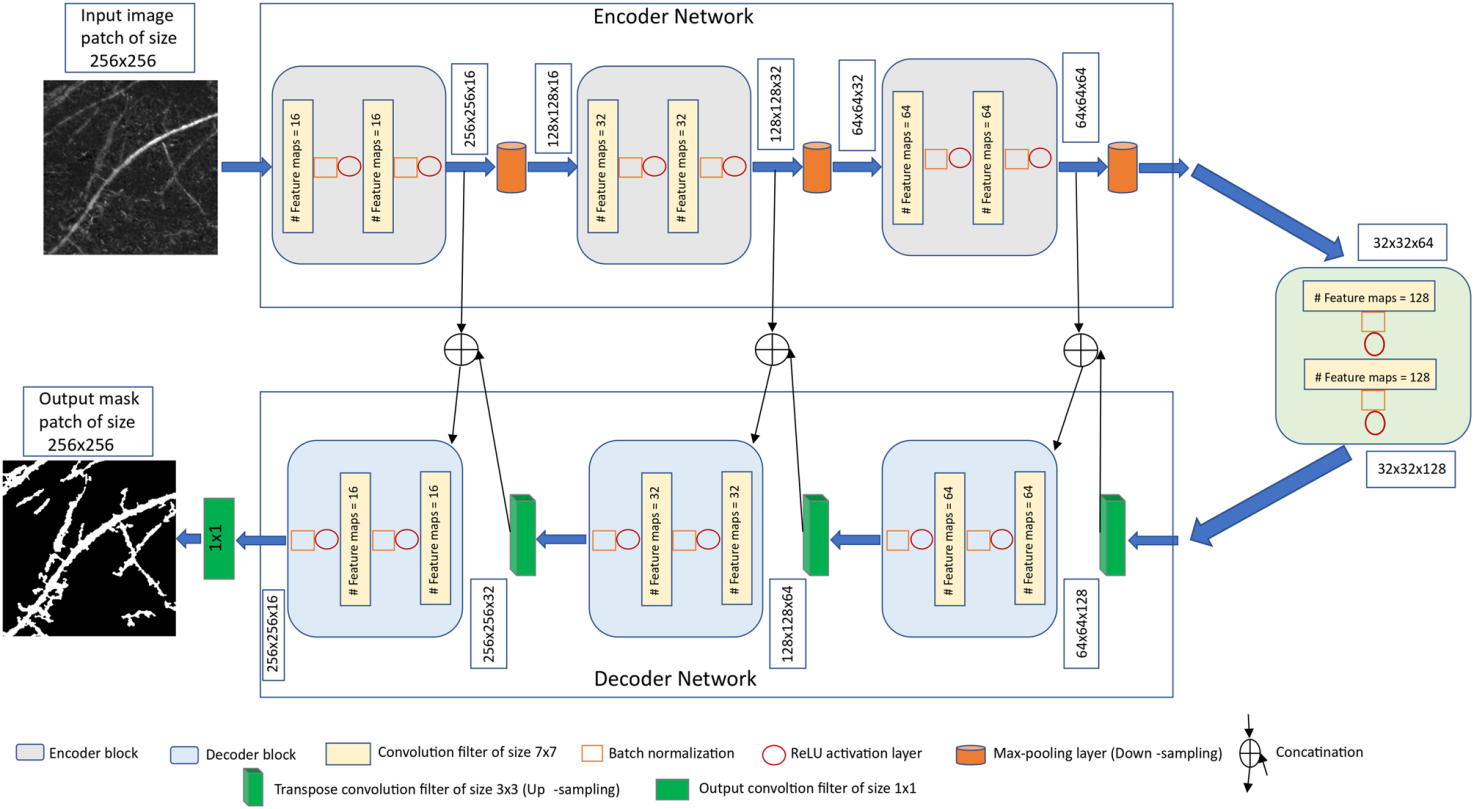
The proposed U-Net architecture for soil-root image segmentation.

Motivated by the encoder–decoder architecture of U-Net, a network framework for soil-root image segmentation was constructed, see Fig. 1. In particular, our network was designed to be trained on patches of input images in original resolution. This was introduced in order to enable model training using larger amount of ground truth data on consumer GPUs while preserving high-frequency image information which otherwise would be lost either by restricting the training set to maximum possible capacity of GPU RAM or by image downscaling. Furthermore, training of CNN on image patches instead of full-size images is known to be more advantageous for learning local features^50^. Therefore, the architecture was designed in such a way that it has input and output layers of the size $256\times 256$. In what follows, the details of network encoder and decoder layers are described.

Encoder network: The encoder network consists of 3 encoder blocks. The first encoder block takes the image patches of size $256\times 256$ as input and produces corresponding feature maps of size ($256\times 256\times 16$) as output. Then the feature maps are forwarded to the second and third encoder blocks to generate further feature maps for the root detection. Each encoder block consists of two convolutional layers to learn feature maps at respective levels, where each convolutional layer consists of $7\times 7$ convolution filter followed by batch normalization^46^ and a non-linear activation function called Rectified Linear Unit (ReLU)^51^. Here, batch normalization improves the network performance and stability by normalizing the feature maps at respective levels^46^. Followed by each encoder block, max-pooling operation using general window size of $2\times 2$^50,52^ is applied for down-sampling the feature maps by half of its original size. This results in aggregate features are generated more efficiently. All three encoders are repeated with varying depth of 16, 32 and 64 to detect diverse root features respectively. The details of each encoder block and corresponding max-pool layers are given in Table 2.

Followed by encoder network, a bridge encoder block without max-pooling layer is applied. This results in 128 feature maps of each size $32\times 32$ are generated.

Decoder network: The output from the bridge encoder ($32\times 32\times 128$) is upsampled using $3\times 3$ transpose convolution with same padding and stride 2. This means size of feature maps ($32\times 32\times 128$) were double to ($64\times 64\times 128$) by applying filter of size $3\times 3$ to all input elements and boarder elements were computed using zero padding. Then the resulting feature map is concatenated with the corresponding encoder feature maps. This results in feature maps of size ($64\times 64\times 256$) are generated. Then it is passed through a convolutional layers like encoder block but having decreasing channel depth of 64. This process is repeated for remaining decoder blocks with decreasing channel depth of 32 and 16. The details of each decoder block and corresponding transpose layer outputs are given in Table 3. Finally, the output of the final decoder block is fed into a convolutional layer of size $1\times 1\times 1$ with “Softmax” activation function^53^ to classify each pixel as root or non-root at the patch level. The output of proposed architecture is a predicted mask of size $256\times 256$ like the input image patch a shown in Fig. 1.

**Table 2:** Details of all encoder blocks and corresponding max-pool layer output.

Encoder block #	Input to encoder block	Convolution filter size	Number of feature maps	Output of encoder block	Input to max-pool	Max-pool output
Block 1	$256\times 256$	$7\times 7$	16	$256\times 256\times 16$	$256\times 256\times 16$	$128\times 128 \times 16$
Block 2	$128 \times 128$	$7 \times 7$	32	$128\times 128\times 32$	$128\times 128\times 32$	$64\times 64\times 32$
Block 3	$64\times 64$	$7 \times 7$	64	$64\times 64\times 64$	$64\times 64\times 64$	$32\times 32\times 64$

**Table 3:** Details of all decoder blocks and corresponding transpose convolutional layers.

Decoder block #	Input to transposed convolution	Output of transposed convolution	Number of decoder blocks	Convolution filter size	Number of feature maps	Output of decoder block
Block 1	$32\times 32\times 128$	$64\times 64\times 128$	$64\times 64\times 128$	$7\times 7$	128	$64\times 64\times 64$
Block 2	$64\times 64\times 64$	$128\times 128\times 64$	$128\times 128\times 64$	$7\times 7$	64	$128\times 128\times 32$
Block 3	$128\times 128\times 32$	$256\times 256\times 32$	$256\times 256\times 32$	$7\times 7$	32	$256\times 256\times 16$

### Performance metrics

To evaluate the performance of the proposed U-Net model during training and testing stage, Dice coefficient (DC)^54^ is used. It measures the area of intersection between the model and ground truth segmentation and its value ranges from 0 to 1, where 1 corresponds to $100\%$ perfect and 0 to false segmentation. The Dice coefficient is defined as:

1 $$\begin{aligned} DC = \frac{2*(P \cap G)}{P \cup G} = \frac{2*\sum _{i}^{N} P_i G_i}{\sum _{i}^{N} P_i + \sum _{i}^{N} G_i}, \end{aligned}$$

where P and G are predicted and ground truth binary images respectively. $P_i$ and $G_i$ are output values 0 and 1 of pixel i in predicted and ground truth binary image respectively. Also, the above equation can be re-written as following:

2 $$\begin{aligned} DC = 2 * \frac{{\text {precision}} * {\text {recall}}}{{\text {precision}} + {\text {recall}}}. \end{aligned}$$

From Eq. (2) it follows that the model would likely overestimate soil pixels and underestimate root pixels in the segmented image, because root images typically contain significantly more background pixels than root pixels. In that case, precision defines the ratio of correctly predicted root pixels to the number of pixels predicted to be root and recall is the ratio of correctly predicted root pixels to the number of actual root pixels in the image.

### Ethical approval

All the protocols involving plants adhered to the ethical guidelines for plant usage were followed while conducting the experiments.

## Experimental setup

### Data and image annotation

Near-infrared (NIR) images of maize plant roots grown in soil were captured by using IPK plant phenotyping system for large plants^17^. Images were taken by one side-view 12MP monochrome camera (UI-5200SE-M-GL, IDS) with chip sensitive in NIR portion of electromagnetic spectrum and suitable distortion-free lens (V1228-MPY). Also, it includes homogeneous infrared LED light source (850 nm) and filters preventing reflections during image acquisition. In brief, plants were grown in rhizopots [$342\times 350$ mm (${\mathrm{W}}\times {\mathrm{L}}$)] filled with the potting substrate (Potgrond P, Klassmann).

**Figure 2:**
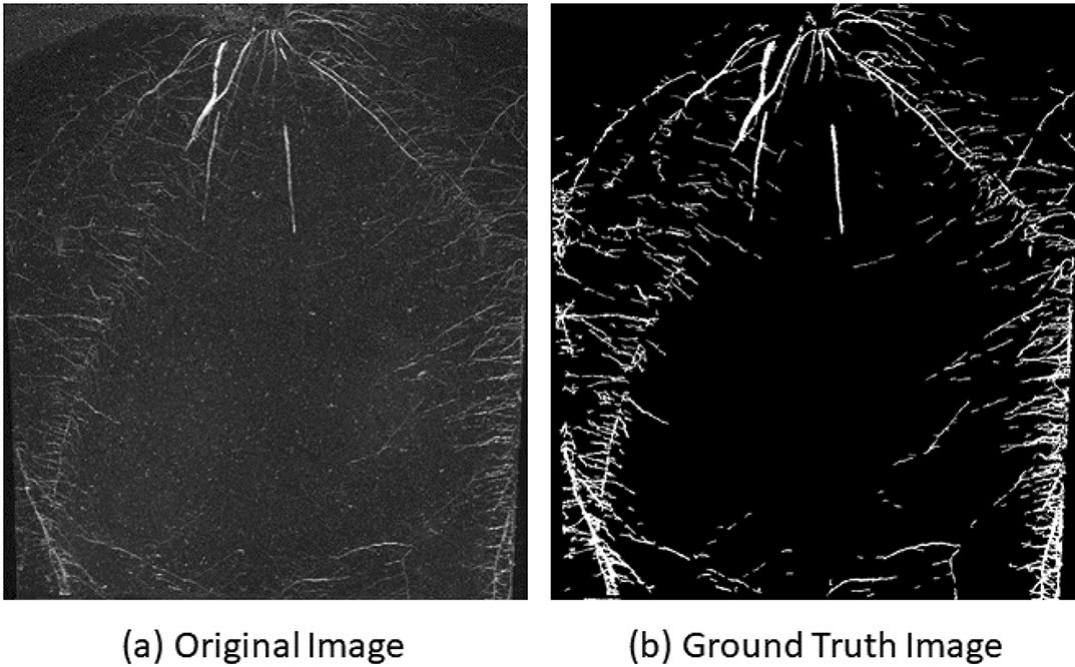
Exemplary root image from IPK plant phenotyping system: (**a**) maize plant roots 28 days after sowing, (**b**) corresponding root segmentation from soil performed using saRIA.

200 greyscale root images of maize plants acquired with the IPK plant phenotyping system were selected for the ground truth segmentation. This labelling task is performed by agronomists using our previously published software for semi-automated root image analysis (saRIA)^26^ which provides an efficient graphical user interface for tuning parameters of image segmentation including intensity threshold, morphology and noise removal to generate an accurate segmentation of roots in soil. The images acquired with the above imaging system have resolution of $2345\times 2665$. A detailed root annotation with saRIA took approximately 5–10 min per image depending on the amount of root pixels in the image. Figure 2 shows an example of IPK plant phenotyping system images and their corresponding binary segmentation using saRIA. This binary mask contains all roots as foreground in white and the remaining pixels as background in black.

To enable application of the proposed model to a broad range of root imaging modalities, the model originally developed for NIR root image segmentation was applied to LED-based rhizotron and ultraviolet (UV) imaging systems^18,26^. In fact, such approach is feasible because root structures in both image modalities exhibit large similarities. The rhizotron system contains a root camera (Allied Vision Prosilica GT 6600) and uses white LED illumination to image the roots growing in soil along plexiglass plates. The UV system contains two monochrome UV-sensitive cameras (UI-5490SE-M-GL, IDS) with two sets of LED illumination panels (UV, 380 nm) in a custom-made imaging box. It is suitable for capturing small plants in transparent pots of size [$77\times 77\times 97$ mm (${\mathrm{W}}\times {\mathrm{L}}\times {\mathrm{H}}$)] filled with the potting substrate (Potgrond P, Klasmann). This system allows non-invasive acquisition of root images in darkness^18^.

### Training

The proposed U-Net model was developed under Python 3.6.1 using TensorFlow^55^ library with Keras API^56,57^. Image processing functions like cropping and morphological functions (dilation, erosion) were implemented using PIL, Numpy^58^ and Scikit-Image^59^ packages. Then the model was trained on Linux operating system (Intel(R) Xeon(R) Gold 6130 CPU @ 2.10GHz) with NVIDIA Tesla P100-PCIE-16GB graphic card.

Images analysed in this work contain both thin and fine root structures that may have only one or few pixels in width. To preserve such fine structures the binary masks were dilated similar to strategy applied in SegRoot^44^. Originally $2345\times 2665$ sized root images of maize plants are analysed step-wise using $256\times 256$ crop masks. Thereby, the original image edges were padded with zeros so that both its width and height are divisible by 256. Hence, original image size is increased to $2560\times 2816$ with zero-padding. Then each image is partitioned into 110 non-overlapping $256\times 256$ crop masks and approximately 20,000 crop masks are generated for all images. However, 2/3 of those cropped masks contain only background structures that contribute to training the network only background appearance. To avoid potential imbalance between plant and non-plant training masks, only cropped regions with both root and background pixels information of the size $256\times 256$ were selected from 182 original images. Then each cropped image is normalized in the range of [0, 1] for feature consistency in the CNN network.

Subsequently, the data set was partitioned into training and validation sets in the ratio of 85:15. The training set is used to optimize the proposed model with Adam optimizer^60^ in such way that the weight parameters improve the model segmentation performance. Also, the initial weights of the networks were defined randomly as proposed by Krizhevsky et al.^61^ with the mean 0 and the standard deviation of 0.05. Here, the model training was initialized for maximum of 200 epochs with 16 number of convolutional channel features and batch size of 128 as per system constraints. Loss functions quantify the unhappiness of our network during training and it defines the difference between predicted output and ground truth generated by saRIA. The result of loss function can be improved by updating weights of the network in an iterative manner. Here, more commonly used “binary cross-entropy loss” function^50^ is used to predict binary class label (i.e., roots and non-roots) at each patch level. This function compares each pixel prediction (0: non-root, 1: root) to the corresponding ground truth pixel and averages all pixels loss for computing total loss of the image. Therefore, each pixel contributes to the overall objective loss function. Then the learning rate of the Adam optimizer^60^ was estimated from a range of reasonable values (0.00001, 0.0001, 0.001, 0.1, 1 and 10) while monitoring the training and validation Dice coefficient of the model.

### Prediction

**Figure 3:**
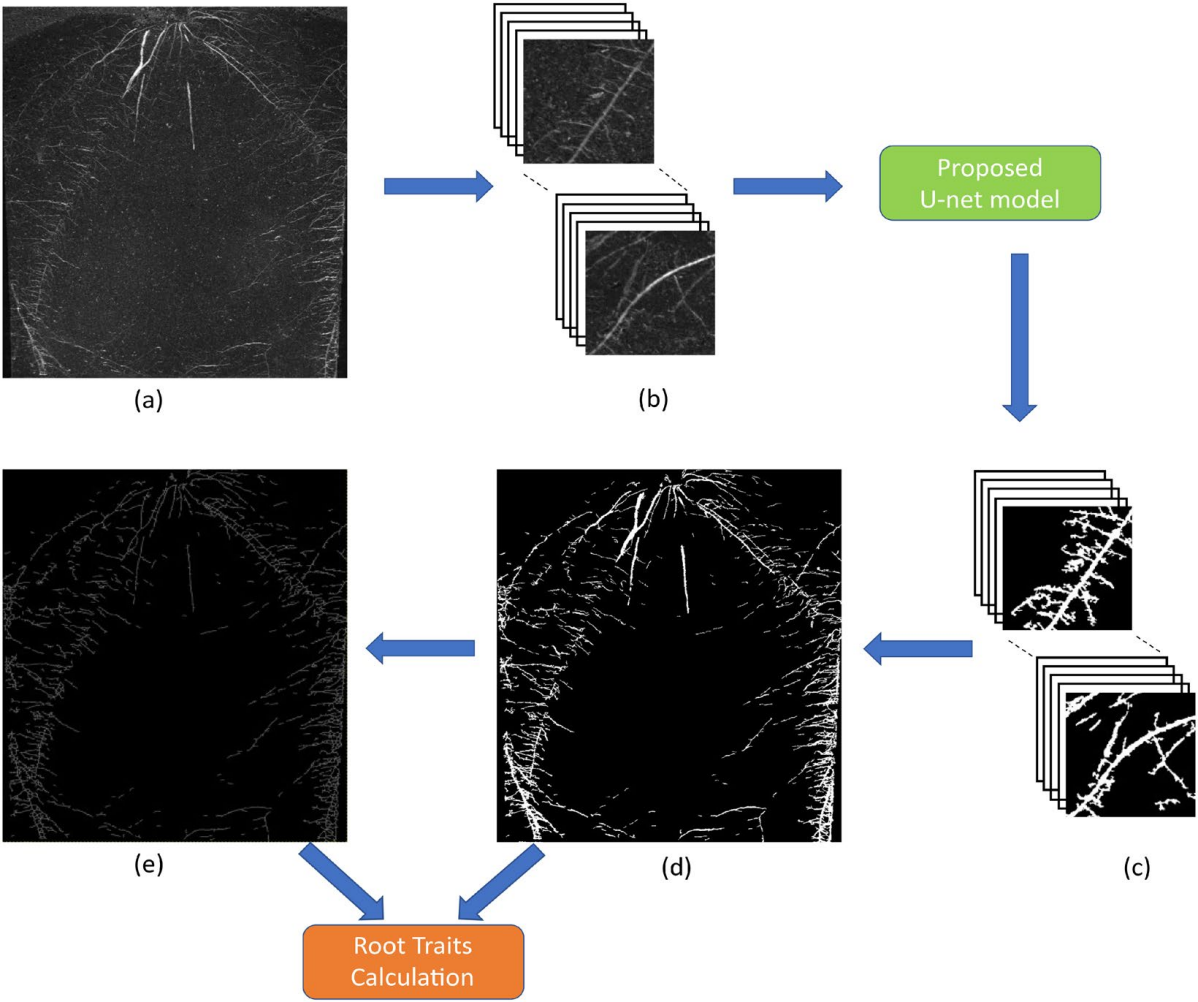
Workflow of the pipeline for image processing and segmentation in faRIA. Green and orange colour boxes represents the operations of image segmentation and trait calculation: (**a**) original image, (**b**) original image patches of size $256\times 256$, (**c**) segmented image patches of size $256\times 256$, (**d**) binary segmentation of original image, (**e**) binary skeleton of (**d**).

As stated in image annotation subsection, the images from IPK plant phenotyping system have the original resolution of $2345\times 2665$, while the proposed U-Net model requires input images of the size $256\times 256$. In the preprocessing stage, zero padding is applied to test images similar as it was done in the training stage. Then non-overlapping $256\times 256$ masks were generated. The model does predictions on these $256\times 256$ masks that are then combined to one single output image. Finally, the zero padded pixels were removed and the segmented image with resolution identical to the original input image was generated. This complete process is dynamic and automatized in the prediction stage as shown in Fig. 3. Since the output layer is given by the Sigmoid activation function, the predicted segmentation is a probability map with values ranging between 0 and 1. Hence the generated probability map was converted to a binary image using threshold T. Here, the root pixels with a relatively high T $\ge$ 0.9 is chosen to avoid misclassification for the soil-root image segmentation. After fully automated segmentation, the proposed model performs phenotyping of segmented root structures similar to saRIA^26^.

In practice, the end-users prefer to have an easy-to-use software solution including the Graphical User Interface (GUI). Therefore, a user-friendly GUI front-end was developed under the MATLAB 2019b environment^62^ to comfortably operate the complex algorithmic framework of faRIA software. Figure 3 shows the complete workflow involved in faRIA for automatic root segmentation and trait extraction. For import of deep learning models trained under Python the MATLAB interoperability routine *importKerasNetwork*^62^ was used. According to specification of this function, the U-Net models trained in Python were exported in the so-called h5 file format, which is supported by the recent versions of MATLAB including 2019b.

In addition to 256 cropped masks, the proposed U-Net model was extended to train on full images. This model has an input and output images of size $1024 \times 1024$ as per our system constraints. So that the original and ground truth images were resized to $1024\times 1024$ using bi-linear interpolation method^63^. Also, the model consist of an additional encoder and decoder blocks with convolution mask of size $5\times 5$ in their respective networks. Therefore, encoder network generates the feature maps from size $1024\times 1024 \times 1$ to $32\times 32\times 128$ and inverse size in the decoder network. To distinguish both networks, the proposed U-Net model on 256 and 1024 masks are named as faRIA:256 and faRIA:1024, respectively.

## Results

### Training and validation of faRIA

**Figure 4:**
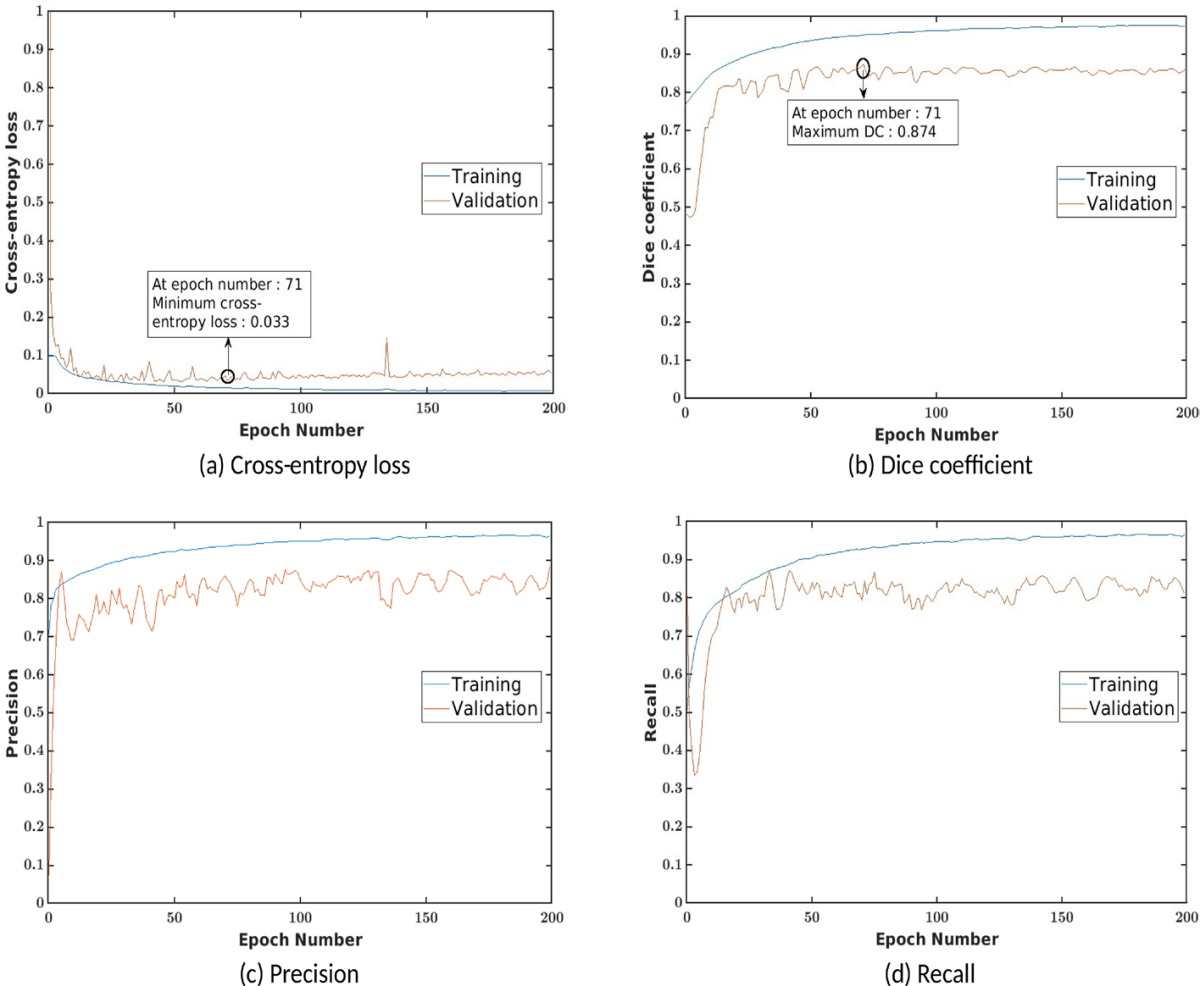
Training and validation performance of the faRIA:256 model over 200 epochs. X- and Y-axes represent the epoch number and performance measure, respectively.

As discussed above, the training and validation of faRIA:256 model was performed on totally 6465 image patches in the ratio of 85:15 between train and test images, respectively. The performance of the trained model is analysed using binary cross-entropy loss, Dice coefficient, precision and recall at each epoch during learning stage of the network. Figure 4 shows the training and validation performance of the faRIA:256 over 200 epochs. It turned out that the training loss (Fig. 4a) was minimized and platen the curve near to zero after epoch number 140. Simultaneously, training DC, precision and recall were maximized and achieved more than 90% of the accuracy from epoch number 100. But generalized performance of the model is measured using validation parameters. Figure 4b explains that the proposed model achieved maximum validation Dice coefficient of 0.874 and minimum validation loss of 0.033 at epoch number 71.

### Evaluation of faRIA versus SegRoot

For comparing the performance of faRIA:256 model with existing tools, SegRoot^44^ was trained on the same image data set. For this purpose, the SegRoot model was trained on $256\times 256$ image blocks for 200 epochs with best practical parameters of depth 5 and width 8 as suggested in Wang et al.^44^. In addition, to validate the performance of proposed model on full image instead of $256\times 256$ blocks (faRIA:256), faRIA:1024 was proposed. The faRIA:1024 model was trained for 200 epochs with training configurations similar to faRIA:256. Tables 4 and 5 show the training parameters and performance measures of the faRIA:256 with respect to SegRoot and faRIA:1024.

**Table 4:** Training parameters of SegRoot, faRIA:1024 and faRIA:256 over 200 epochs.

Training parameter	SegRoot-8-5	faRIA:1024	faRIA:256
Learning rate	0.01	0.001	0.001
Batch size	128	3	128
Epochs	200	200	200

**Table 5:** Training performance of SegRoot, faRIA:1024 and faRIA:256 over 200 epochs.

Validation measure	SegRoot-8-5	faRIA:1024	faRIA:256
Cross-entropy loss	0.374	0.043	0.033
Dice coefficient	0.666	0.888	0.874
Precision	0.652	0.901	0.849
Recall	0.735	0.824	0.846

**Figure 5:**
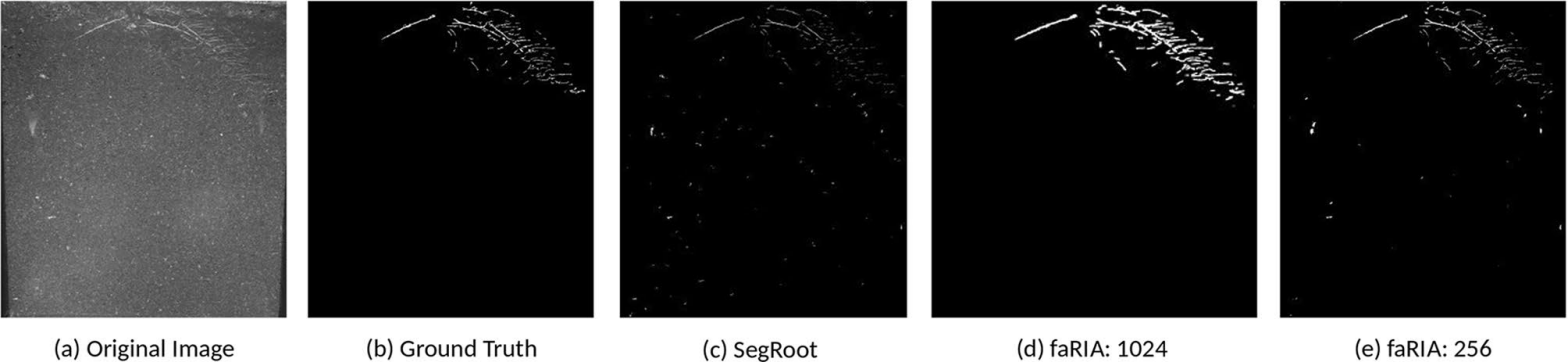
Segmentation performance: (**a**) original image, (**b**) ground truth segmentation by saRIA, (**c**) SegRoot with DC: 0.42, (**d**) faRIA:1024 with DC: 0.44, (**e**) faRIA:256 with DC: 0.83.

**Figure 6:**
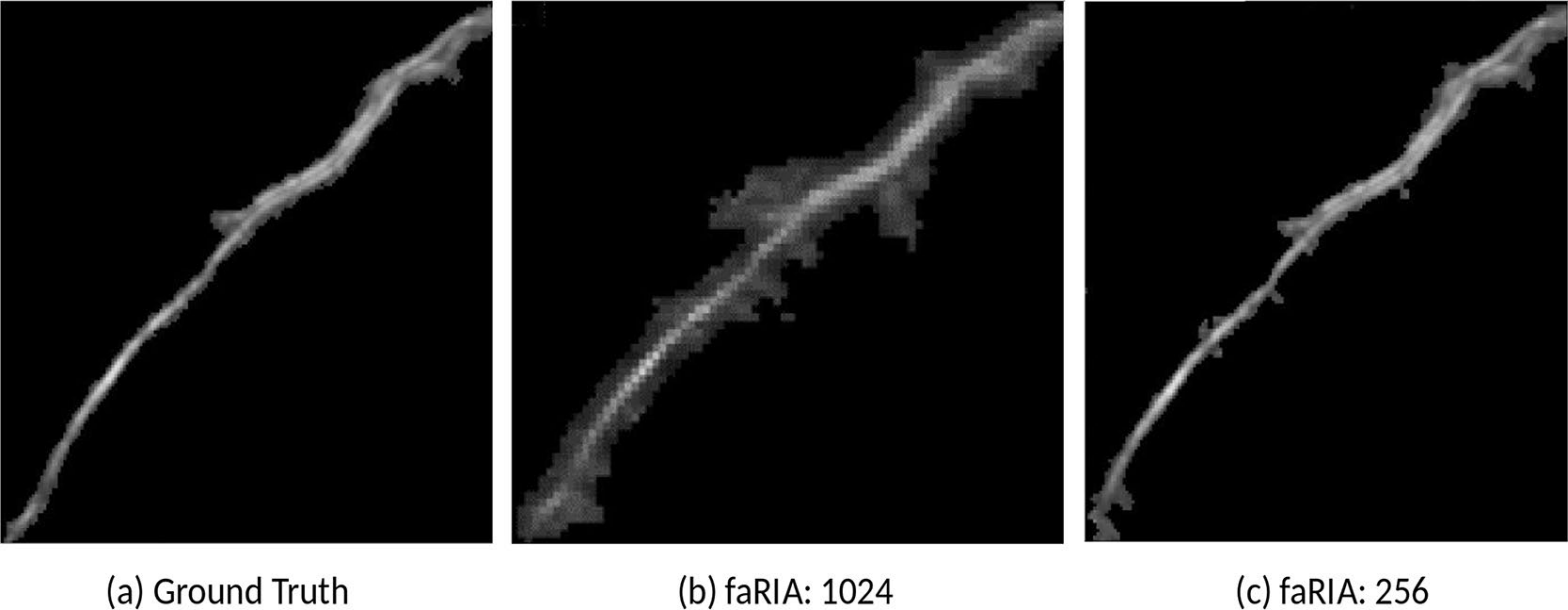
Segmentation artefacts: (**a**) original root structure, (**b**) faRIA:1024: noisy segmentation of (**a**) at root edges, (**c**) faRIA:256: noise-free segmentation of (**a**).

Followed by training performance, an exemplary performance of above three models on test image was performed, see in Fig. 5. Thereby, the faRIA:256 model showed the DC of 0.83 whereas SegRoot and faRIA:1024 achieved 0.42 and 0.44 respectively. Also, the presence of marginal artefacts in faRIA:1024 and faRIA:256 compared to ground truth are shown in Fig. 6.

### Segmentation of further image modalities

**Figure 7:**
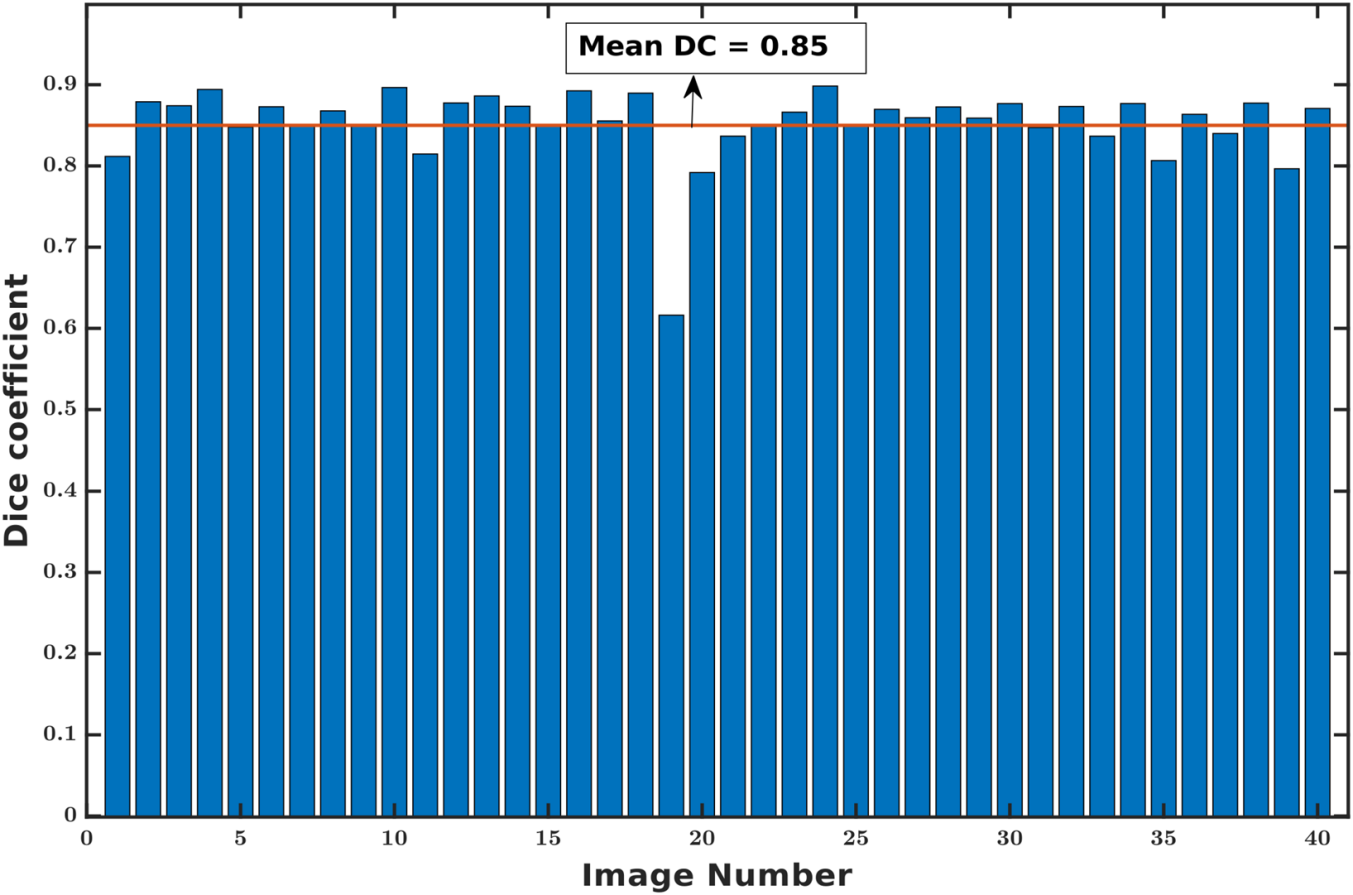
Dice coefficient of faRIA:256 over 40 barley soil-root images from rhizotron imaging system. The orange line represents the mean DC value.

**Figure 8:**
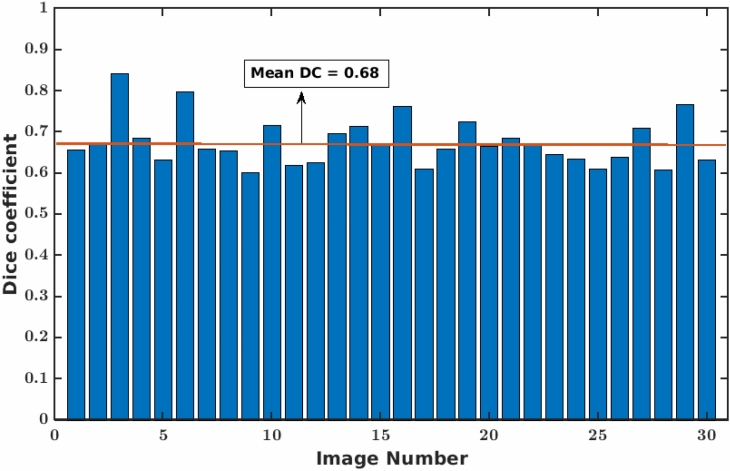
Dice coefficient of faRIA:256 over 30 arabidopsis soil-root images from UV imaging system. The orange line represents the mean DC value.

**Figure 9:**
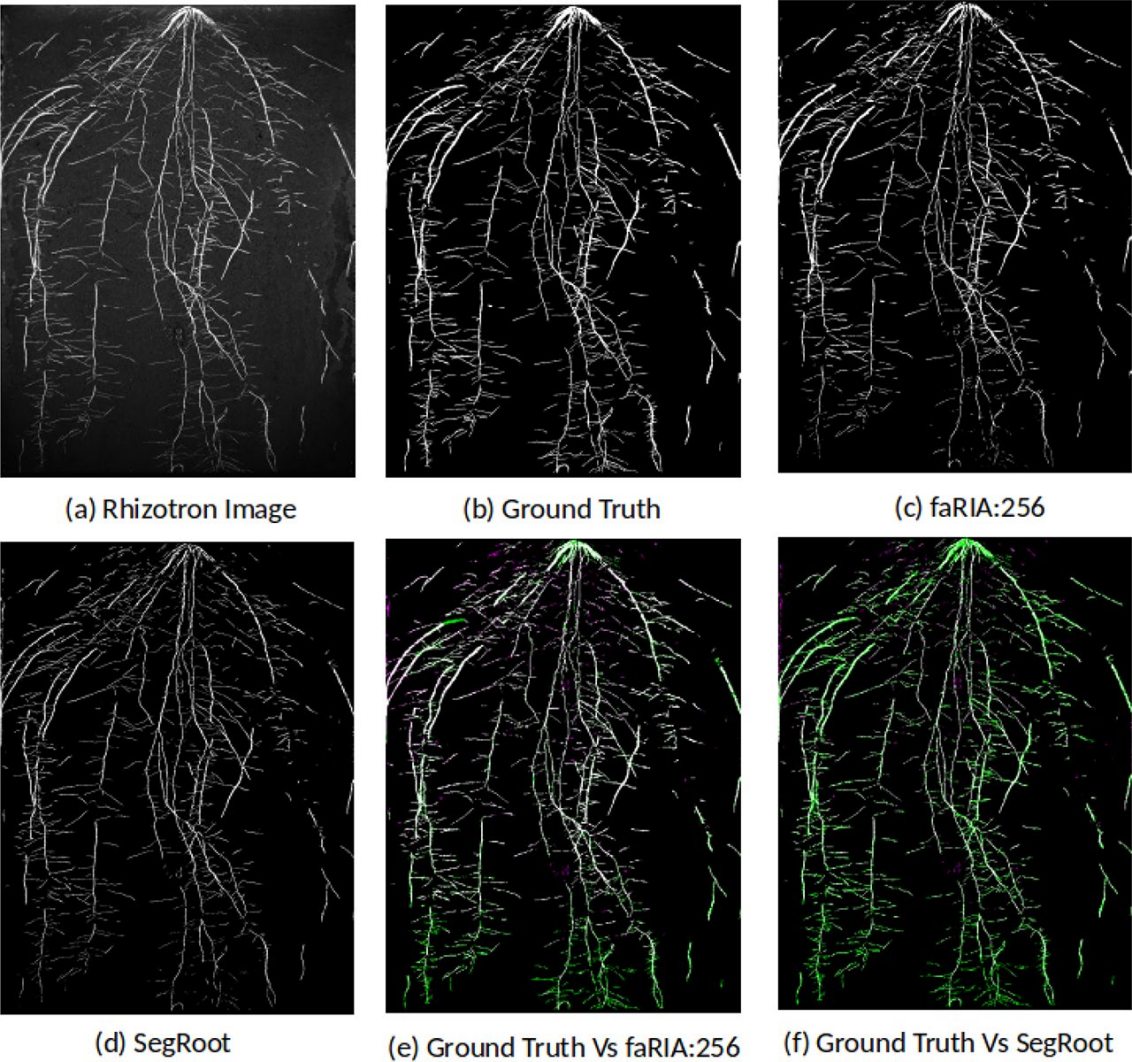
Applicability of faRIA on rhizotron soil-root images: (**a**) image of barley plant roots at lateral stage, (**b**) ground truth segmentation generated by saRIA, (**c**) predicted segmentation using faRIA:256, (**d**) predicted segmentation using SegRoot, (**e**) overlay of faRIA prediction on ground truth with DC = 0.87, (**f**) overlay of SegRoot prediction on ground truth with DC = 0.73. Green, pink and white colour pixels represents false negatives, false positives and correctly segmented pixels in the predicted image with respect to ground truth respectively.

**Figure 10:**
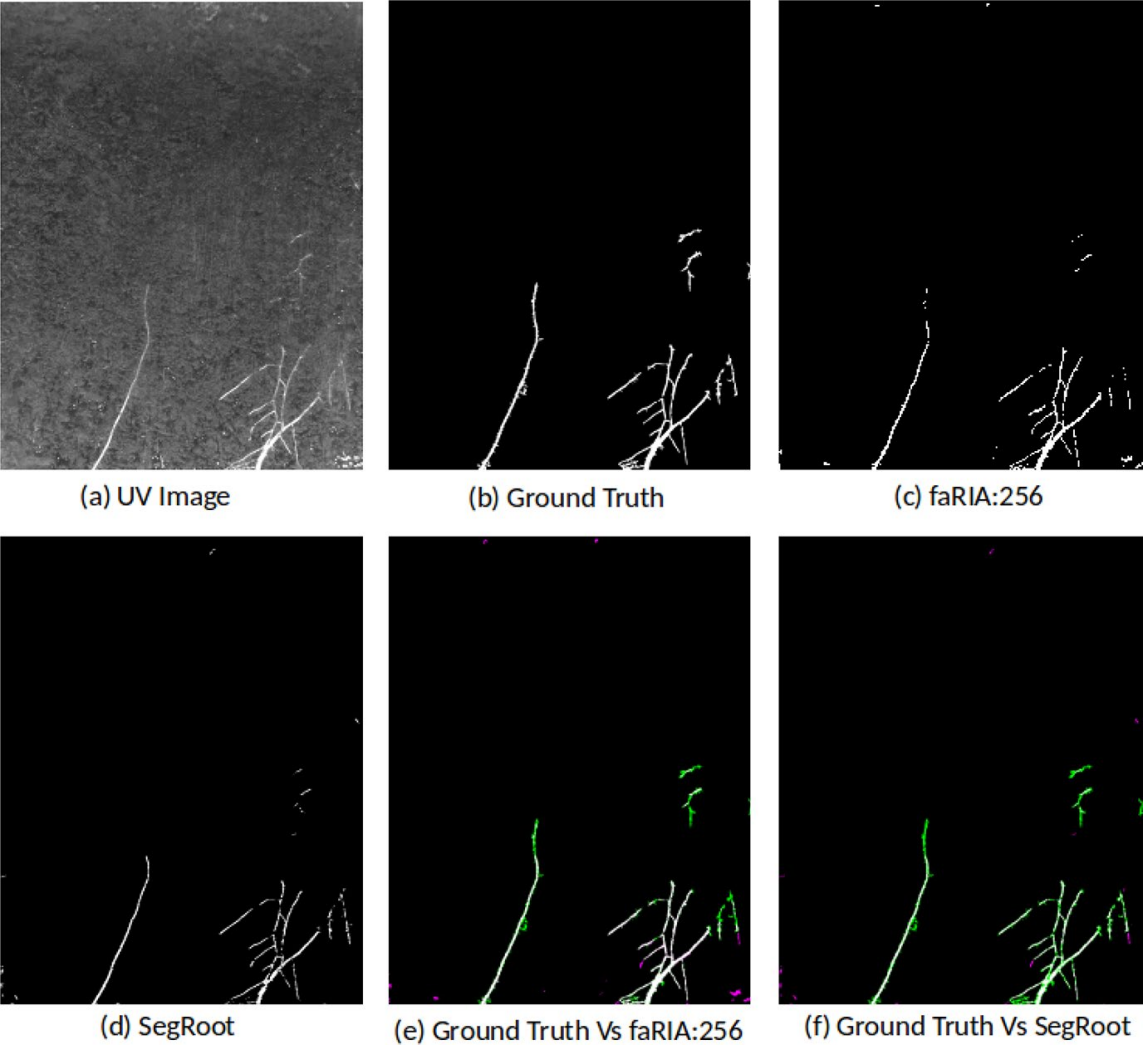
Applicability of faRIA on UV soil-root images: (**a**) image of arabidopsis plant roots from UV imaging system, (**b**) ground truth segmentation generated by saRIA, (**c**) predicted segmentation using faRIA:256, (**d**) predicted segmentation using SegRoot, (**e**) overlay of faRIA prediction on ground truth with DC = 0.80, (**f**) overlay of SegRoot prediction on ground truth with DC = 0.67. Green, pink and white colour pixels represents false negatives, false positives and correctly segmented pixels in the predicted image with respect to ground truth respectively.

The faRIA:256 model originally trained on maize plant roots from IPK plant phenotyping system is applied to LED-based rhizotron and UV imaging systems for the root segmentation from soil. Figures 7 and 8 shows the DC of faRIA:256 model over 40 barley and 30 arabidopsis root images from rhizotron and UV imaging system and achieved mean DC of 0.85 and 0.68 respectively. An exemplary segmentation of rhizotron (image number 4 in Fig. 7) and UV image (image number 6 in Fig. 8) are shown in Figs. 9a–c,e and 10a–c,e respectively. Here, the faRIA:256 model resulted DC of 0.87 and 0.79 for rhizotron and UV image compared to the ground truth generated by saRIA respectively. In addition, the performance of the SegRoot on same rhizotron and UV image compared to the ground truth is shown in the Figs. 9d,f and 10d,f respectively. Here, false negative (green) and false positive (pink) pixels represents the undetected and falsely classified root pixels in the predicted segmentation compared to the ground truth.

### Evaluation of phenotypic traits versus saRIA

**Figure 11:**
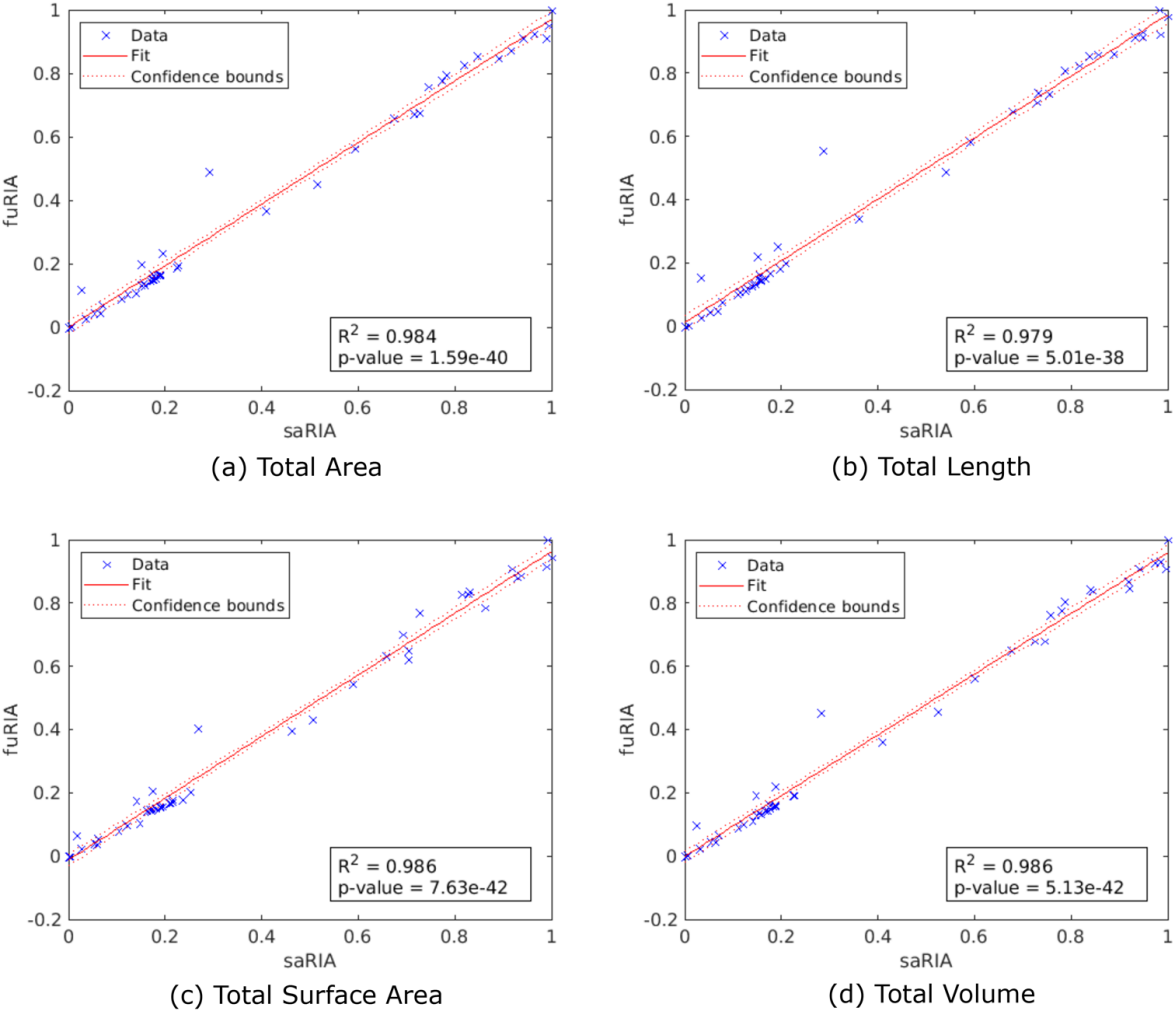
Correlation between root traits calculated using semi-automated saRIA (x-axis) and fully-automated faRIA (y-axis) image segmentation. Each point represents a trait value estimated from one of 40 soil-root images from rhizotron imaging system. The red colour solid line and dotted lines represent a fitted curve and 95% confidence bounds, respectively. The $R^2$ value indicates good conformity between saRIA and faRIA results of image segmentation and trait calculation.

In addition to the segmentation performance, phenotyping characterization obtained with faRIA are also evaluated in comparison to saRIA. Here, correlation coefficient of determination $R^2$ and significance level *p* value are used to measure the percent of the faRIA calculated traits that are close to the ground-truth (from saRIA) and model validation respectively. Figure 11 shows the correlation between the saRIA (x-axis) and faRIA (y-axis) outputs for four traits where each point denotes one particular image out of 40 barley root images from rhizotron imaging system. Out of 75 traits, only four important traits for root biomass calculation are presented for faRIA evaluation. They are total root area, total root length, total root surface area and total root volume. Further information on definition of traits is included in the Supplementary Information, see Table S1. Figure 11 shows that correlations between traits calculated with saRIA and faRIA are highly significant and exhibit $R^2$ values greater than 0.98, 0.97, 0.98 and 0.98 and *p* values 1.59e−40, 5.01e−38, 7.63e−42, and 5.13e−42, respectively.

### Graphical user interface and runtime

**Figure 12:**
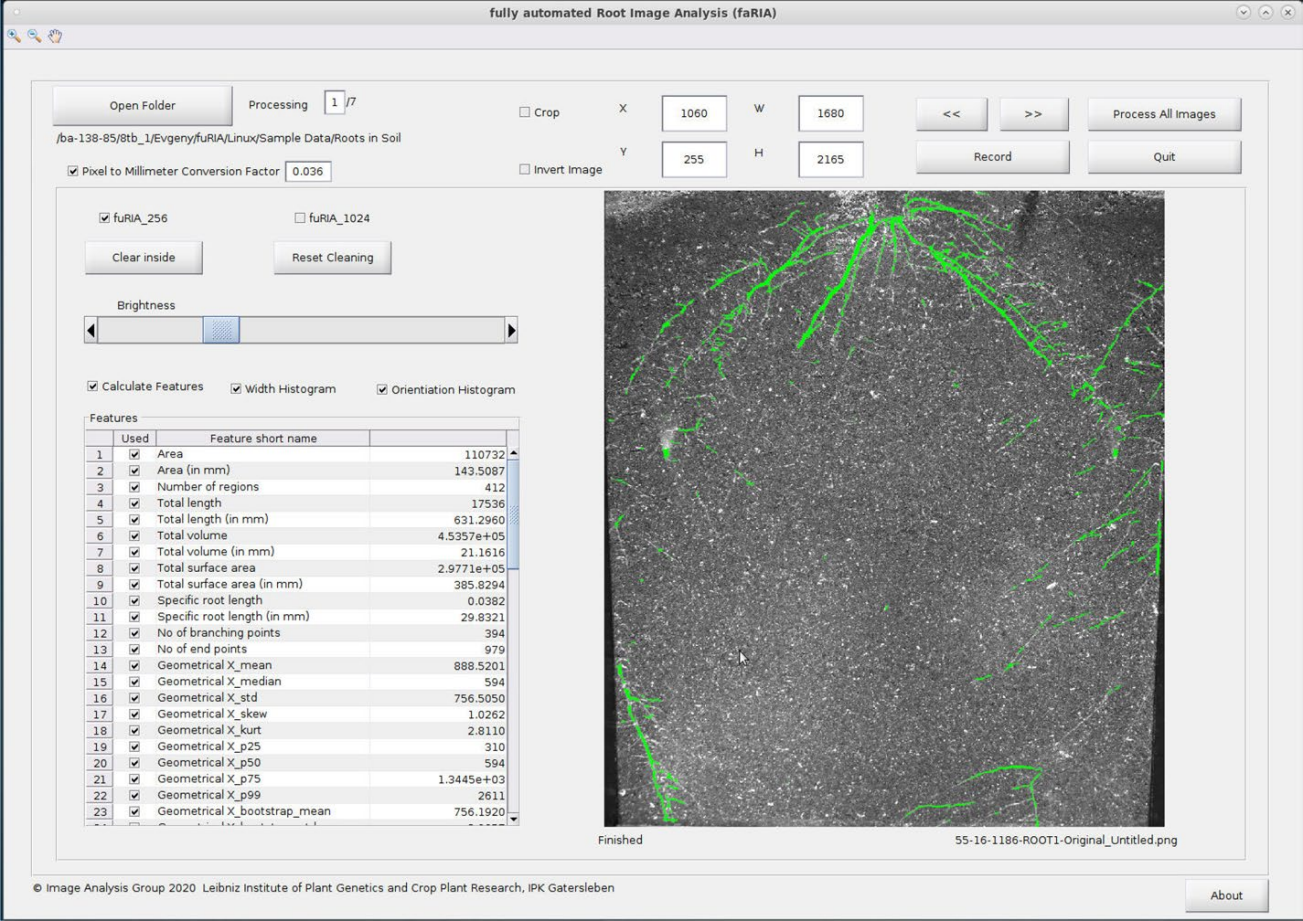
Graphical user interface of faRIA. Green colour pixels represent root regions automatically segmented by the U-Net model.

Figure 12 shows the GUI of faRIA software which is freely available as a precompiled executable program from https://ag-ba.ipk-gatersleben.de/faria.html. In addition to fully automated image segmentation, faRIA calculates 75 root traits that are categorized into 12 feature groups named area (number of root pixels), number of disconnected root objects, total length, surface area, volume, number of branching and ending points, statistical distribution (mean, median, standard deviation, skewness, kurtosis, percentile and bootstrap) of root geometry in horizontal and vertical direction, width, orientation and convex-hull. In the present release, the phenotyping module of faRIA is identical to our saRIA software^26^. Further information on definition of traits is included in the Supplementary Information, see Table S1.

The faRIA software provides users with an option to select faRIA:256 or faRIA:1024 model depending on image quality, time and accuracy. The faRIA software can analyse a single image or large image data set to automatically detect and extract multiple root traits. Regarding timing performance, the faRIA segmentation, root tracing and trait calculation all together take, in average, 80 s using faRIA:256 and 15 s using faRIA:1024 models to process and analyse a 6-megapixel (cropped) image on a system with Intel(R) Xeon(R) Gold 6130 CPU @2.10GHz. Therefore, faRIA:1024 can process at least 3 times faster than faRIA:256 for root image analysis.

## Discussion

Our experimental results on different plant species from different imaging systems have demonstrated a remarkable accuracy of an adopted U-Net model for fully automated soil-root image segmentation. During the training stage, the faRIA:256 model achieved nearly zero loss and $\ge$ 95% of accuracy measured by the Dice coefficient (DC) crossover 200 epochs, see Fig. 4. By application to the test images, the best performance was found at the epoch number 71 with the maximum DC of 0.874 and minimum loss of 0.033. For larger number of epochs, validation error was just marginally higher. However, the precision and recall are contrasting each other at low DC epochs, and both achieved maximum at epoch number 71. Therefore, the network weights and optimization parameters at epoch number 71 are adopted as the best model for soil-root image segmentation.

The performance of the faRIA:256 model was compared with the SegRoot. From the summary in Table 5, it is evident that faRIA:256 is significantly outperforming the SegRoot on our data set with improving the cross-entropy loss by the factor 10 and DC by 20%, respectively. We draw this results back to the fact that the SegRoot model transfers only max-pooling indices (i.e., location of feature maps) from encoder to decoder for feature concatenation and reconstruction, whereas our U-Net model transfers complete feature map information (i.e., both location and pixel values) to the decoder. This leads to detection of both primary and secondary low contrast roots with the improved DC in comparison to the SegRoot, see Fig. 5. However, more information required for U-Net makes the decoder path expensive and requires more memory (9.47 MB) than the SegRoot (1.49 MB).

In addition to the faRIA:256 model, which was trained on $256\times 256$ patches of original large root images, the performance of proposed U-Net architecture was reformulated on full images and validated with images downscaled to the size of $1024\times 1024$ due to our hardware limitations using the faRIA:1024 model. While both faRIA:1024 and faRIA:256 models demonstrated a comparable accuracy in the training stage, faRIA:256 exhibits more balanced performance between precision and recall than faRIA:1024. This imbalance is cased by the pixels of intermediate intensity on the boundary between the soil and root regions that correspond to average values calculated by downscaling. Pixels of intermediate intensities lead to false positive detection (Fig. 5b). In particular, it is the case by segmentation of thin root structures in downscaled images using the faRIA:1024 model.

Since roots and background regions exhibit similar structural properties in images of different modalities and plant species, our model originally trained on NIR maize roots images could also be applied to barley and arabidopsis roots from LED-rhizotron and UV imaging systems, respectively. For rhizotron images it achieved the minimum accuracy of 80% for all images with exception of the image number 19 in Fig. 7. The overall mean DC = 0.85 indicates a fairly accurate segmentation of rhizotron images. The exceptional image with the number 19 exhibit low DC due to the presence of high intensity noise similar to root structures. Moreover, our model preserves the root thickness and continuity in the secondary roots compared to the SegRoot as shown in Fig. 9e,f. This results in DC of rhizotron image 0.87 is higher than the SegRoot 0.73.

The application of faRIA on UV images, the accuracy of the faRIA:256 model ranged between 60 and 83% with the mean DC = 0.7, see Fig. 8. A relatively low DC for some UV images is due to the presence of diverse artefacts including low contrast between the root architecture and heterogeneous soil regions, in-homogeneous scene illumination (i.e., vertical intensity gradient). This results in inaccurate segmentation (pink colour pixels) of low contrast structures and false detection of high intensity background structures as shown in Fig. 10. However, faRIA:256 achieved the continuity in the root segmentation along the contrast varying root structures with DC of 0.80 (Fig. 10e) whereas SegRoot results in discontinues root structures with DC of 0.67 (Fig. 10f). Therefore, approximately 80% of the root pixels were correctly detected by faRIA:256 compared to the ground truth. Further examples of NIR, rhizotron and UV root image segmentation for juvenile or adult plants are in the Supplementary Information (see Figs. S1–S6).

Furthermore, a direct comparison between phenotypic traits calculated with semi-automated (saRIA) and fully automated (faRIA) approaches shows a highly significant correlation which indicates that root image segmentation and phenotyping using faRIA as practically as good as human-supervised one.

Further, investigations with extended and/or augmented image data are required to improve the accuracy of segmentation of other root images that were not included in the original training set. On the other hand, it cannot be excluded that training of dedicated models with a narrow focus on a particular type of imaging modality and image structures could be a more reliable strategy to achieve more accurate results.

## Conclusion

Automated segmentation and analysis of a large amount of structurally heterogeneous and noisy soil-root images is a challenging task which solution is highly demanded in quantitative plant science. Here, we present an efficient GUI-based software tool for fully automated soil-root image segmentation which relies on the U-Net CNN architecture trained on a set of 6465 masks derived from 182 manually segmented soil-root images. The proposed algorithmic framework is capable to efficiently segment root structures of different size, shape and contrast with higher accuracy of DC = 0.87 in comparison to the state-of-the-art solutions (SegRoot: DC = 0.67). Our experimental results showed that the model trained with representative patches of root and background structures enables consideration of a larger amount ground truth data than original full-size images. Thereby, the faRIA:256 model trained on smaller size masks outperforms the larger mask model (faRIA1024) with respect to the overall precision and recall by comparison with ground truth data. In addition to NIR maize root images that were originally used for CNN model training, the faRIA tool can also be applied to other imaging modalities and plants species that exhibit similar structural properties of root and background regions. In addition to root image segmentation, faRIA calculates a number of useful phenotypic traits that in our experimental studies were shown to exhibit a significant correlation ($R^2=0.98$) with the ground truth traits. While the present CNN framework was predominantly trained with regular soil-root images, further investigations are required to address such challenging problems as segmentation of roots overlaid with a large scale noise (for example, due to water condensation) or filling artificial gaps in the root system that occur due to inhomogeneous scene illumination. Possible approaches to addressing these problems include, for example, appropriate augmentation of the training data set and/or alternative CNN models.

## Supplementary Information

Supplementary Information.
