# Supplementary material for: Fully-automated root image analysis (faRIA)
Source: Sci Rep. 2021 Aug 6;11:16047. doi: 10.1038/s41598-021-95480-y (PMC8346561; doi:10.1038/s41598-021-95480-y)
Supplement: Supplementary file 1 — Supplementary Information. [file 41598_2021_95480_MOESM1_ESM.pdf]

Supplementary Material

Fully-automated Root Image Analysis (faRIA), N. Narisetti*, M. Henke, C. Seiler, A. Junker, J.Ostermann, T. Altmann, E. Gladilin*.

Table S1. Root Traits: The description of estimated root system architecture traits in faRIA software.

**Trait Name**	**Description**
Area	Number of root pixels in the image
Number of Regions	Number of disconnected root objects in the image
Total Length	The sum of major axis length of each root object approximated by fitting ellipse to the root object
Total Volume (V)	The sum of local volume at each root object of skeleton approximated by tubular shape whose average radius is estimated from image<br><br>$V=\sum_{i=0}^{n} \pi r_i^2$<br>Where r_i is the average radius of ith root component in the image.
Total Surface Area (SA)	The sum of surface area at each root object of skeleton approximated by tubular shape whose average radius is estimated from image<br><br>$SA=\sum_{i=0}^{n} 2\pi r_i$<br>Where r_i is the average radius of ith root component in the image.
Specific Root Length	The ratio of total length and total volume of roots in the image.
Number of Branching Points	The total number of branches in the root skeleton
Number of End Points	The total number of end points in the root skeleton
Geometrical X_mean, Y_mean	The mean value of root pixels distribution in horizontal and vertical direction
Geometrical X_median, Y_median	The median value of root pixels distribution in horizontal and vertical direction
Geometrical X_std, Y_std	The standard deviation of root pixels distribution in horizontal and vertical direction

Geometrical X_skew, Y_skew	The skewness of root pixels distribution in horizontal and vertical direction
Geometrical X_kurt, Y_kurt	The kurtosis of root pixels distribution in horizontal and vertical direction
Geometrical X_p25, Y_p25	The 25 percentile of root pixels distribution in horizontal and vertical direction
Geometrical X_p50, Y_p50	The 50 percentile of root pixels distribution in horizontal and vertical direction
Geometrical X_p75, Y_p75	The 75 percentile of root pixels distribution in horizontal and vertical direction
Geometrical X_p99, Y_99	The 75 percentile of root pixels distribution in horizontal and vertical direction
Geometrical X_bootstrap_mean, Y_bootstrap_mean	The mean value of bootstrapping root pixels distribution in horizontal and vertical direction
Geometrical X_bootstrap_stdev, Y_bootstrap_stdev	The median value of bootstrapping root pixels distribution in horizontal and vertical direction
Width mean	Average root diameter
Width median	Median root diameter
Width std	Standard deviation of the root diameter
Width skew	Skewness of root diameter
Width kurt	Kurtosis of root diameter
Width p25	25 percentile of root diameter
Width p51	50 percentile of root diameter
Width p75	75 percentile of root diameter
Width p99	99 percentile of root diameter
Width bootstrap_mean	The mean value of bootstrapping root pixels width
Width bootstrap_median	The median value of bootstrapping root pixels width
Orientation mean	Average root Orientation
Orientation median	Median root Orientation
Orientation std	Standard deviation of the root Orientation
Orientation skew	Skewness of root Orientation
Orientation kurt	Kurtosis of root Orientation
Orientation p25	25 percentile of root Orientation
Orientation p50	50 percentile of root Orientation
Orientation p75	75 percentile of root Orientation
Orientation p99	99 percentile of root Orientation
Orientation bootstrap_mean	The mean value of bootstrapping root pixels orientation

Orientation bootstrap_median	The median value of bootstrapping root pixels orientation
SeedAngle mean	Mean orientation of root pixels with respect to root originating seed point in RSA
SeedAngle median	Median orientation of root pixels with respect to root originating seed point in RSA
SeedAngle skew	Skewness of root pixels with respect to root originating seed point in RSA
SeedAngle kurt	Kurtosis of root pixels with respect to root originating seed point in RSA
SeedAngle p25	25 percentile of root pixels with respect to root originating seed point in RSA
SeedAngle p50	50 percentile of root pixels with respect to root originating seed point in RSA
SeedAngle p75	75 percentile of root pixels with respect to root originating seed point in RSA
SeedAngle p99	99 percentile of root pixels with respect to root originating seed point in RSA
SeedAngle bootstrap_mean	The mean value of bootstrapping root pixels seed orientation
SeedAngle bootstrap_stdev	The median value of bootstrapping root pixels orientation
ConvexHull area	Area of convex-hull of RSA
ConvexHull width	Width of convex-hull of RSA
ConvexHull height	Height of convex-hull of RSA
ConvexHull specific_area	The ratio of convex-hull area and actual area of RSA

Figure S1. Example of NIR root image segmentation for juvenile Maize plant using the faRIA:256 model.

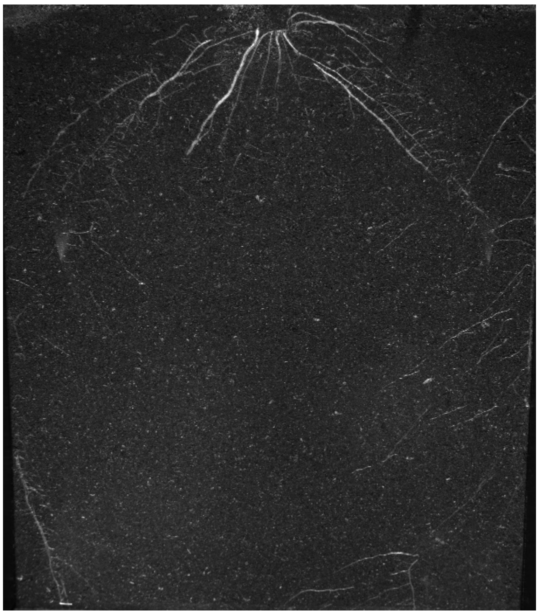

(a) Original Image

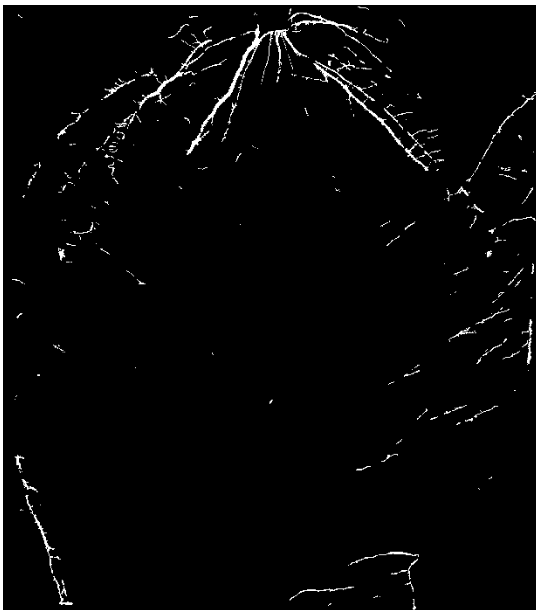

(b) Segmented Image

Figure S2. Example of NIR root image segmentation for adult Maize plant using the faRIA:256 model.

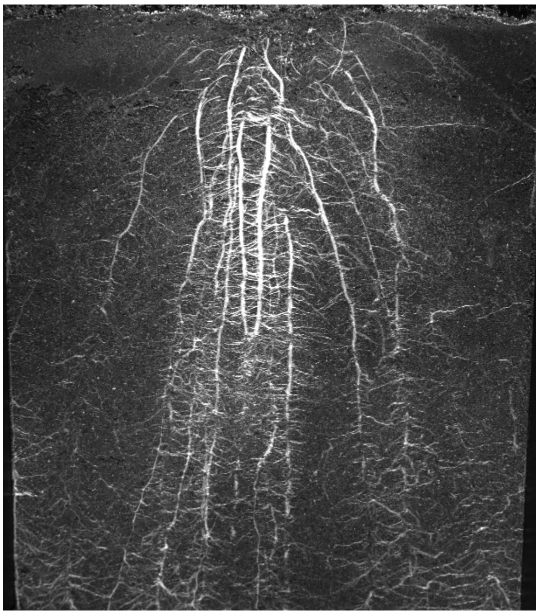

(a) Original Image

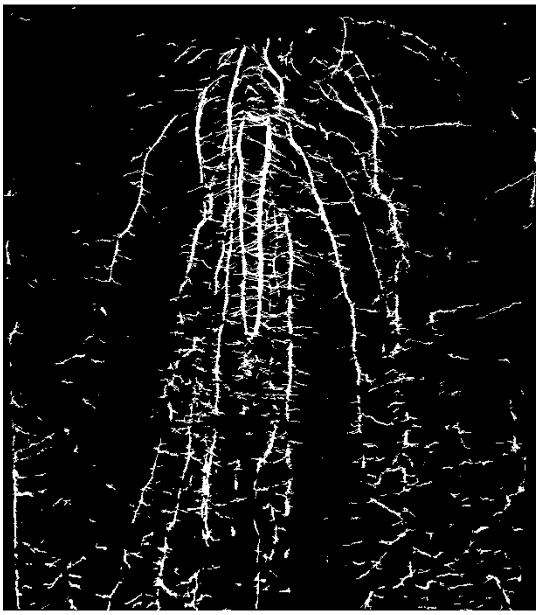

(b) Segmented Image

Figure S3. Example of Rhizotron root image segmentation for juvenile Barley plant using the faRIA:256 model.

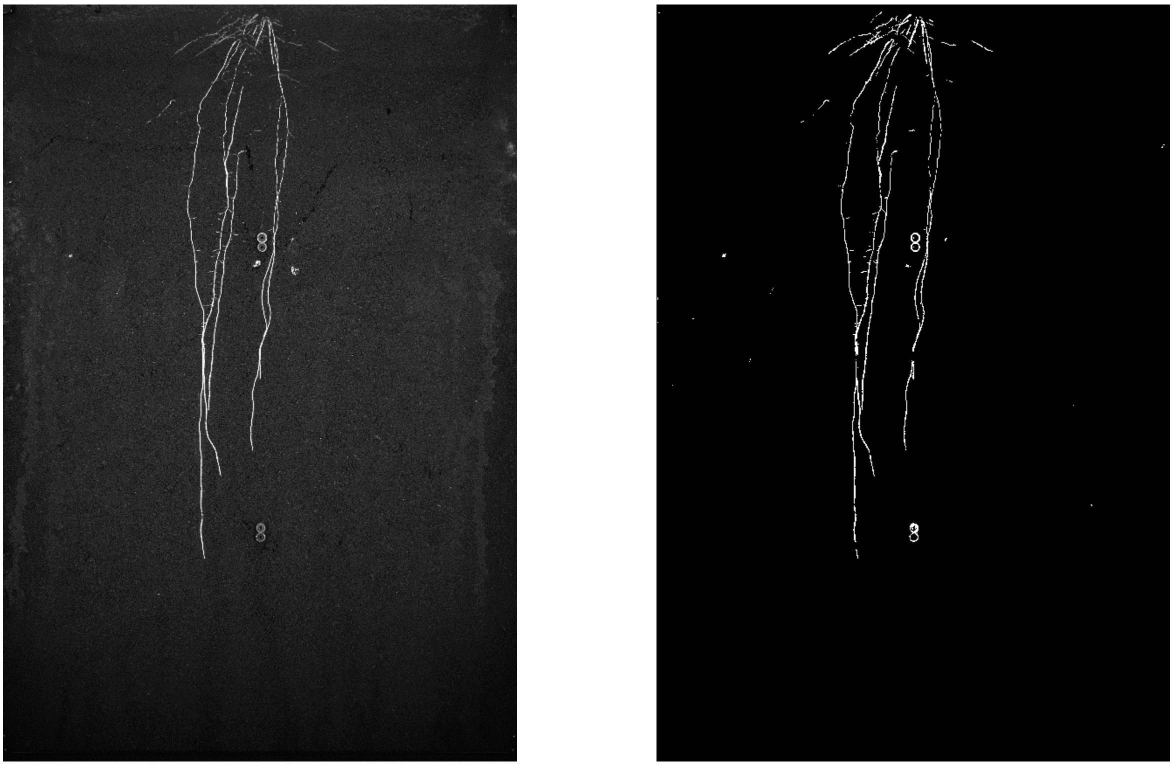

(a) Original Image (b) Segmented Image

Figure S4. Example of Rhizotron root image segmentation for adult Barley plant using the faRIA:256 model.

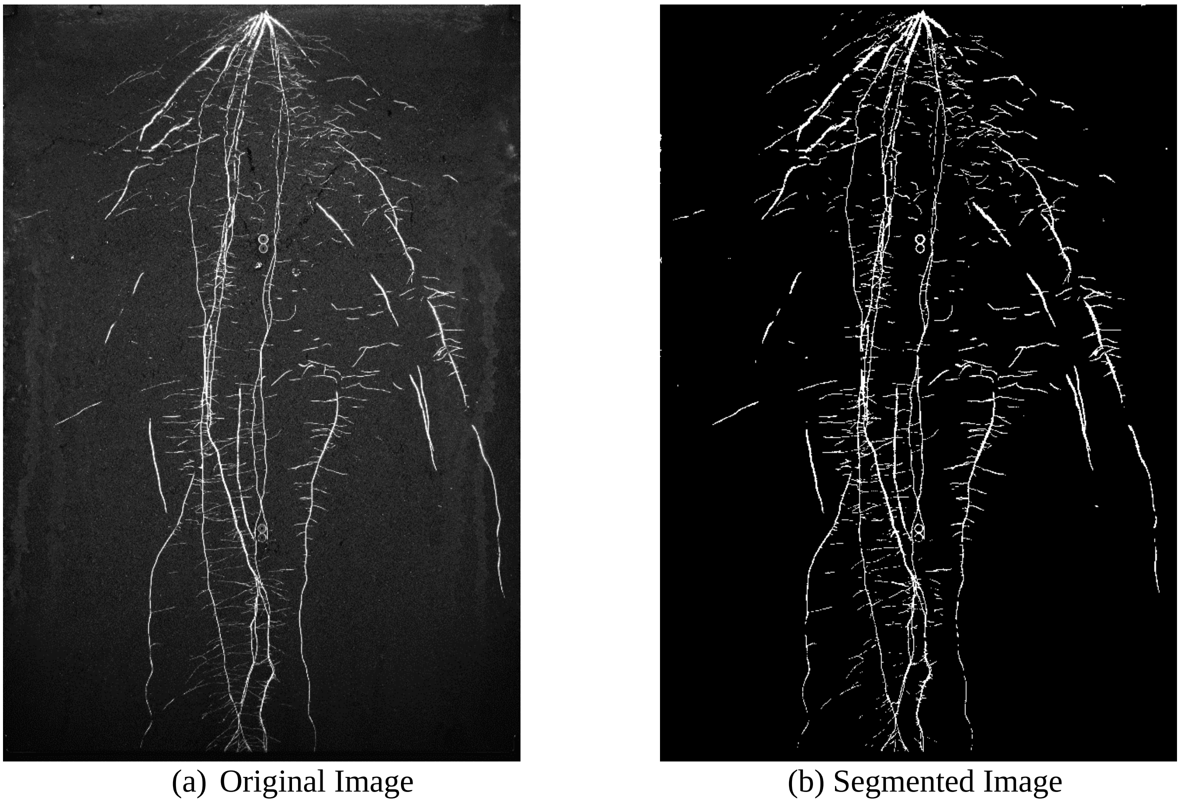

(a) Original Image (b) Segmented Image

Figure S5. Example of UV root image segmentation for juvenile Arabidopsis plant using the faRIA:256 model.

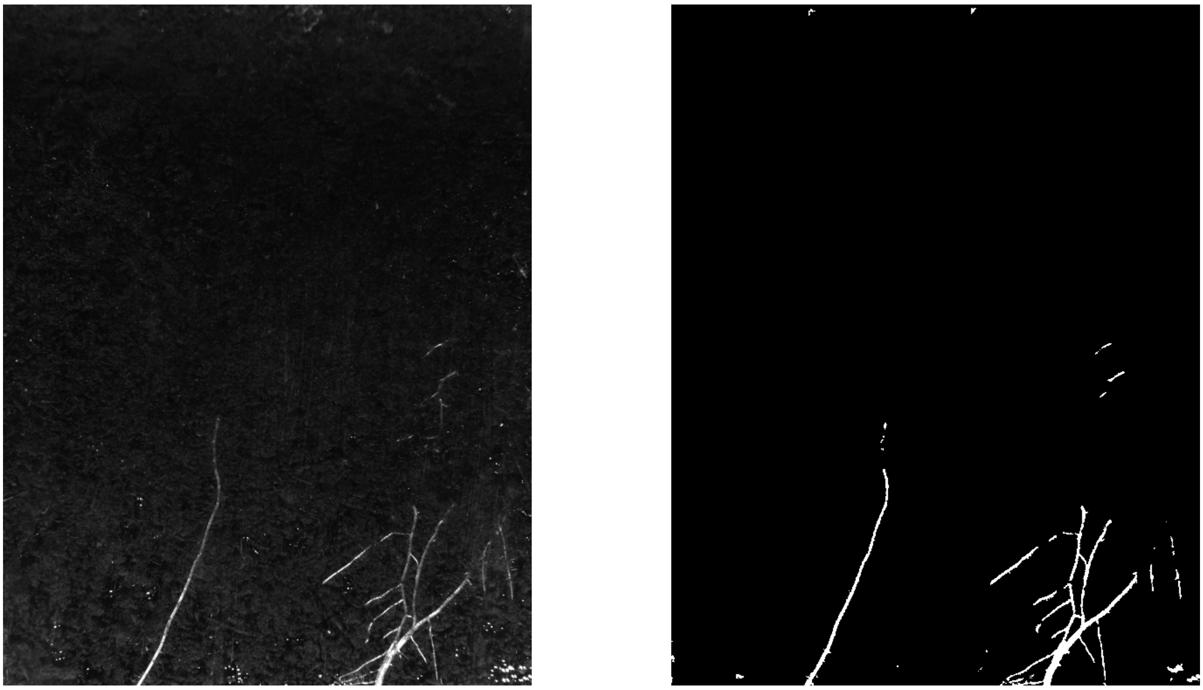

(a) Original Image (b) Segmented Image

Figure S6. Example of UV root image segmentation for adult Arabidopsis plant using the faRIA:256 model.

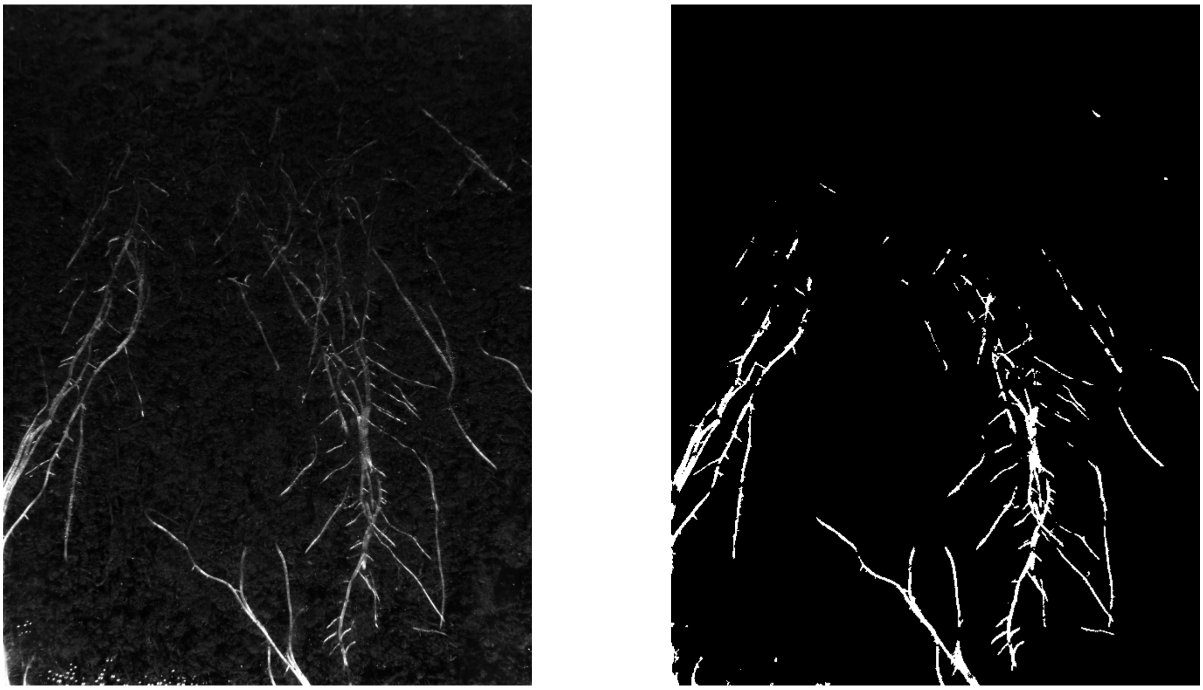

(a) Original Image (b) Segmented Image
